# Complementary Use of Presepsin with the Sepsis-3 Criteria Improved Identification of High-Risk Patients with Suspected Sepsis

**DOI:** 10.3390/biomedicines9091076

**Published:** 2021-08-24

**Authors:** Jong Eun Park, Beomki Lee, Sun Joo Yoon, Chi-Min Park, Chul Won Jung, Myung-Ju Ahn, Hyung-Doo Park, Sung Yeon Hwang, Tae Gun Shin, Eun-Suk Kang

**Affiliations:** 1 Department of Emergency Medicine, Samsung Medical Center, Sungkyunkwan University School of Medicine, Seoul 06351, Korea; jongeun7.park@samsung.com (J.E.P.); sygood.hwang@samsung.com (S.Y.H.); 2 Department of Emergency Medicine, College of Medicine, Kangwon National University, Chuncheon 24289, Korea; 3 Department of Laboratory Medicine and Genetics, Samsung Medical Center, Sungkyunkwan University School of Medicine, Seoul 06351, Korea; beomki.lee@skku.edu (B.L.); nayadoo@hanmail.net (H.-D.P.); 4 Department of Internal Medicine, Seoul National University Bundang Hospital, Seongnam 13620, Korea; yoonsunjoo@gmail.com; 5 Department of Critical Care Medicine, Samsung Medical Center, Sungkyunkwan University School of Medicine, Seoul 06351, Korea; dr99.park@samsung.com; 6 Department of Surgery, Samsung Medical Center, Sungkyunkwan University School of Medicine, Seoul 06351, Korea; 7 Division of Hematology-Oncology, Department of Medicine, Samsung Medical Center, Sungkyunkwan University School of Medicine, Seoul 06351, Korea; leukemia1@skku.edu (C.W.J.); silk.ahn@samsung.com (M.-J.A.)

**Keywords:** presepsin, sepsis, septic shock, mortality

## Abstract

Presepsin has been proposed as an early indicator for diagnosis and prognosis in sepsis. We aimed to evaluate the prognostic accuracy of presepsin levels and additional value for identifying high-risk patients when taken together with the current sepsis criteria. This was a single-center, prospective, observational study of patients with suspected sepsis. The primary outcome was 28-day mortality. The prognostic performance of presepsin was evaluated by the area under the receiver operating characteristic curve (AUC), according to the sepsis definition using the Sequential Organ Failure Assessment (SOFA) score change (delta SOFA ≥ 2) and lactate level ≥ 2 mmol/L. A total of 755 patients were included. The AUC of presepsin for predicting 28-day mortality was 0.747. Presepsin showed adequate prognostic accuracy regardless of the delta SOFA score or lactate level. High presepsin levels (>755 pg/mL) showed an independent association with 28-day mortality (adjusted odds ratio: 5.17), and significant differences in mortality were observed, even in patients with non-sepsis low lactate level. Compared with a single criterion of the delta SOFA score or lactate, the addition of the high presepsin criterion significantly increased discrimination. Presepsin showed fair prognostic performance regardless of the clinical sepsis criteria. Complementary use of presepsin with the Sepsis-3 criteria may identify more high-risk septic patients and provide useful prognostic information.

## 1. Introduction

The worldwide incidence of sepsis-related morbidity and mortality remains high [1]. To improve clinical outcomes, early identification of septic patients at high risk of mortality is critical. Nonetheless, an accurate and timely diagnosis of sepsis is challenging. In terms of pathophysiology, sepsis is defined as a life-threatening organ dysfunction caused by a dysregulated host response to infection [2]. A change in the Sequential Organ Failure Assessment (SOFA) score of two or more is the clinical surrogate criteria of sepsis and is associated with mortality of approximately 10% [2].

Along with clinical scoring, biomarker measurements can help physicians predict outcomes of patients with suspected infection and detect sepsis syndrome. Various circulating biomarkers have shown prognostic value in many studies, but few markers have been used in clinical practice due to the controversial issues in clinical value [3]. Among them, procalcitonin (PCT), lactate, and C-reactive protein (CRP) have been widely used for risk assessments of critically ill septic patients [4]. However, these biomarkers also have limitations, including the nonspecific nature of sepsis and the fact that no single biomarker is sufficient to represent all aspects of sepsis [5].

Presespin, also known as soluble CD14 subtype (sCD14-ST), is a fragment of CD14 that is released from activated monocytes following recognition of lipopolysaccharide (LPS) [6,7]. It has been suggested as an early indicator for diagnosis and prognosis in sepsis [6,8]. Compared to CRP and PCT, presepsin may be secreted earlier and can reach its peak faster [6,8,9]. Therefore, presepsin is expected to be a promising biomarker that could identify patients with sepsis who are at high risk for poor prognosis more quickly and sensitively. However, there have been conflicting results regarding the clinical value of presepsin [4,10].

In this study, the primary aim is to evaluate the prognostic accuracy of presepsin in patients with suspected sepsis. We also investigated whether presepsin could be used complementarily with the current sepsis definition and lactate to identify high-risk patients.

## 2. Materials and Methods

### 2.1. Study Design and Population

This was a single-center, prospective, and observational study of patients with suspected sepsis who visited Samsung Medical Center—a 1989-bed, university-affiliated, tertiary care referral hospital located in Seoul, Korea. The study period was July 2020 through September 2020. We prospectively included consecutive patients according to the following inclusion criteria: age ≥18 years old; suspected sepsis with procalcitonin measurement and blood culture test; and visited the emergency department (ED) or admitted to either the hematology-oncology department or the intensive care unit (ICU). We tested presepsin from 778 patients using residual plasma after procalcitonin measurement. Among the collected samples tested for presepsin, samples that fulfilled either of the following conditions were excluded: presepsin measured more than two h after measuring procalcitonin, or duplicate samples during a hospitalization period lasting less than one week. If there were multiple results for a patient, only the first test performed was incorporated in the analysis. The patients without blood cultures at the same time were also excluded. The Institutional Review Board of Samsung Medical Center approved this study (IRB No. SMC-2019-05-170). Informed consent was waived because this study was anonymous using residual samples.

### 2.2. Measurement of Presepsin

Samples were collected with a plasma separating tube (PST) containing sodium heparin anticoagulant (Vacutainer PST Tube 8.0 mL, #367964; Becton Dickinson [BD], Sunnyvale, CA, USA). Procalcitonin (PCT) was tested with Elecsys BRAHMS PCT assay (Roche, Basel, Switzerland) on Cobas e801 (Roche, Mannheim, Germany). After testing PCT, the residual samples were subjected to presepsin measurement with the PATHFAST^TM^ assay kit (LSI Medience Corporation, Tokyo, Japan) and PATHFAST^TM^ analyzer (LSI Medience Corporation, Tokyo, Japan) within two h. The PATHFAST^TM^ presepsin assay is a cartridge-type, fully-automated enzyme immunoassay system that consists of magnetic particles coated with mouse monoclonal antibodies and alkaline phosphatase (ALP)-conjugated rabbit polyclonal antibodies [11]. The immunocomplex resulting from presepsin binding with the two antibodies generates luminescence after incubation with the chemiluminescent substrate [11]. The test requires 100 μL as the minimum sample volume and takes about 17 min [11].

Prior to measuring the subjects’ presepsin levels, laboratory performance validation of the PATHFAST^TM^ assay kit and analyzer was conducted. Imprecision was assessed with two different levels of quality control (QC) materials measured in duplicate runs, twice a day, for five days. The results showed within-run coefficient of variation (CV) and within-laboratory CV below 5%. Linearity was evaluated with five different levels of verification control measured in four replicates. The coefficient of determination (R2) was 0.998, and the 95% confidence interval (CI) for the slope of the linear regression model ranged from 0.904 to 1.073.

Stability over time at room temperature was also evaluated in order to determine the exclusion criterion. A total of 45 samples evenly distributed along the measuring range (low, <300 pg/mL; medium, ≥300 pg/mL and <1000 pg/mL; and high, ≥1000 pg/mL) were tested 0, 1, 2, and 3 h after testing PCT. The mean value for each sample was calculated, and the difference between each test and the mean value was determined. While the difference was less than 20% of the mean value until 2 h after testing PCT, the difference between the two tests exceeded 20% of the mean value after 3 h. In our institute, PCT is a stat test which should be reported within 60 min after sample collection; thus, it was determined that presepsin tests performed more than 3 h after sample collection should be excluded.

### 2.3. Data Collection and Definitions

Along with procalcitonin and presepsin, clinical data including demographic data, comorbidities, suspected infection focus, vital signs, laboratory tests, and blood cultures were obtained. The nearest values from the time of procalcitonin measurement were used, and the SOFA score was calculated with them. Missing values were not imputed. According to the Sepsis-3 definition, sepsis was defined as an acute change in total SOFA score (delta SOFA) ≥2 points consequent to the infection [2]. The baseline SOFA score was assumed to be 0 if it was unknown. Septic shock was defined as sepsis with persisting hypotension requiring vasopressors to maintain mean arterial pressure (MAP) ≥65 mmHg and a serum lactate level > 2 mmol/L despite adequate volume resuscitation. The primary outcome was 28-day mortality.

### 2.4. Statistical Analysis

Standard descriptive statistics were used for all variables including baseline demographics and outcomes. Categorical variables were presented as numbers with percentages and compared using a chi-square test. Continuous variables were presented as medians and interquartile ranges (IQRs) and compared using the Wilcoxon rank-sum test. The prognostic performance of presepsin for 28-day mortality was assessed as the area under the receiver operating curve (AUC), sensitivity, specificity, positive predictive value (PPV), and negative predictive value (NPV). We used a nonparametric approach for the comparison of two AUCs [12]. The Youden index was used to determine the optimal cut-off value for presepsin. According to the cut-off level, patients were categorized into the high presepsin group or the low presepsin group.

A multivariable logistic regression model was developed to assess variables related to 28-day mortality. The goodness-of-fit of the final logistic regression model was assessed using the Hosmer–Lemeshow test. The results were described as adjusted odds ratio (aOR) with a 95% confidence interval (CI). A *p*-value less than 0.05 was considered to indicate a statistically significant difference. STATA 15 (Version 15.0, STATA Corporation, College Station, TX, USA) was used for statistical analysis.

## 3. Results

### 3.1. Baseline Characteristics

**Figure 1:**
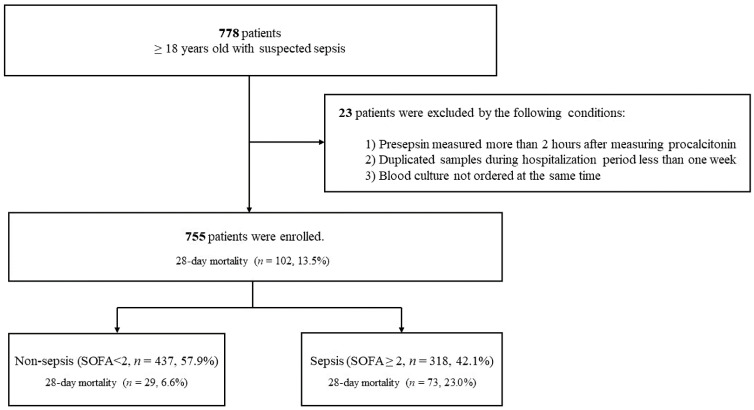
Study flowchart.

A total of 755 patients with suspected sepsis were included (Figure 1). There was no confirmed case of coronavirus disease 2019 (COVID-19) in this study. By hospital location, the proportions of patients were 72.5% (*n* = 547) in the ED, 20.3% (*n* = 153) in the general ward, and 7.3% (*n* = 55) in the ICU. The 28-day mortality was 13.5% (*n* = 102). The baseline characteristics of all patients and a comparison between survivors and non-survivors are shown in Table 1. The median age was 63.2 years (IQR 51.9–72.1), and 56.3% (*n* = 425) were men. The most common focus of infection was respiratory infection (24.5%), followed by urinary tract infection (15.5%) and gastrointestinal infection (14.2%). Characteristics such as older age, male sex, malignancy, respiratory infection focus, and number of patients with sepsis were more frequently observed among non-survivors than in survivors. The SOFA score, lactate, CRP, and procalcitonin at enrollment were significantly higher in non-survivors than survivors.

The number of non-sepsis, sepsis, and septic shock patients was 437 (57.9%), 274 (36.3%), and 44 (5.8%), respectively, and the 28-day mortality in each of the three groups was 6.6%, 20.1%, and 40.9%, respectively. Baseline characteristics of the patients with non-sepsis, sepsis, and septic shock are shown in Supplementary Table S1.

### 3.2. Presepsin Levels

**Figure 2:**
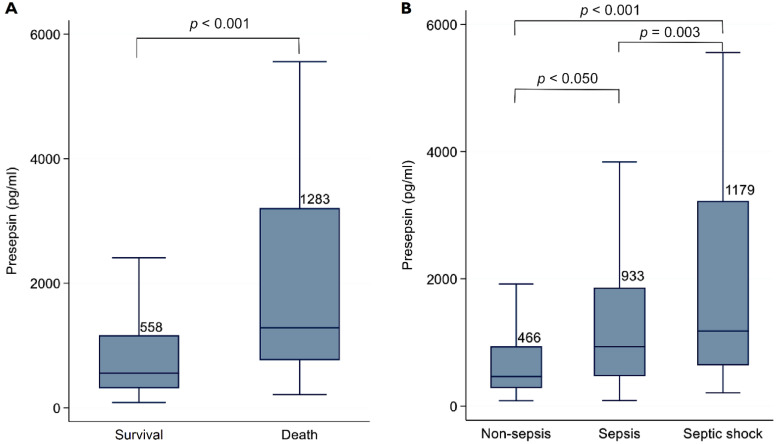
Comparison of presepsin levels according to (**A**) survival and (**B**) the sepsis criteria.

The median presepsin level was significantly higher in non-survivors than in survivors (1283 pg/mL (IQR, 765–3208) vs. 558 pg/mL [IQR, 315–1164], *p* < 0.001) (Figure 2). The level of presepsin was highest in septic shock (1179 pg/mL (IQR, 643–3225)), followed by sepsis (933 pg/mL (IQR, 472–1861)) and non-sepsis (466 pg/mL (IQR, 284–938)), and there was a statistically significant difference in each group. Among sepsis patients, the presepsin level of septic shock patients was also significantly higher than in patients without shock (*p* = 0.003). The presepsin levels were still significantly different according to survival and the sepsis criteria, regardless of the presence of monocytopenia (<500 × 10^3^/μL). In patients with monocytopenia, the median presepsin levels were 570 pg/mL (IQR, 295–1189) in survivors vs. 1438.5 pg/mL (IQR, 889–4311) in non-survivors (*p* < 0.001), and 441.5 pg/mL (IQR, 266–937) in non-sepsis patients vs. 1054 pg/mL (IQR, 505–1906) in sepsis patients vs. 1246.5 (IQR 732–3181) in patients with septic shock (*p* = 0.028). Presepsin level was also significantly higher in patients with bacteremia than in those without (1214 pg/mL (IQR, 687–2713) vs. 562 pg/mL (IQR, 315–1173), *p* < 0.001). The difference in presepsin levels between gram-positive and gram-negative bacteremia was not statistically significant. According to the Youden index, the optimal presepsin cut-off point for predicting 28-day mortality was 755 pg/mL.

### 3.3. Multivariable Logistic Regression Analysis for 28-Day Mortality

The results from univariable and multivariable logistic regression analysis for 28-day mortality are presented in Table 2. In multivariable logistic regression analysis, we adjusted predefined variables including age, sex, infection focus, SOFA score, lactate level, procalcitonin level, and comorbidities with a significant difference between survivors and non-survivors. A high presepsin level, over 755 pg/mL, was significantly associated with 28-day mortality in both the univariable analysis (Odds ratio; OR 5.61, 95% CI 3.44–9.16, *p* < 0.001) and the multivariable model that adjusted for confounding factors (aOR 5.17, 95% CI 2.92–9.16, *p* < 0.001). The presence of malignancy, respiratory infection, delta SOFA score ≥ 2, and lactate ≥ 2 mmol/L were also significantly associated with 28-day mortality.

### 3.4. Discriminating Ability of Presepsin for 28-Day Mortality Based on Delta SOFA Score and Lactate Level

**Figure 3:**
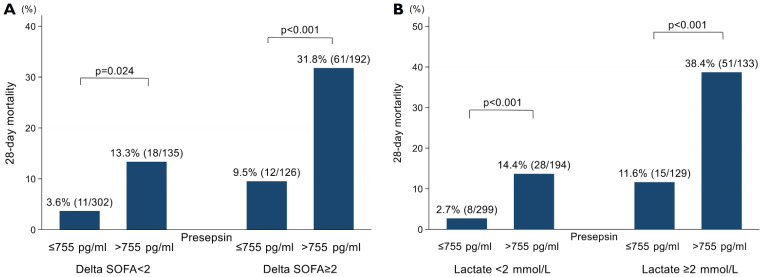
Comparison of 28-day mortality according to the level of presepsin based on (**A**) delta sofa score and (**B**) lactate value, respectively. SOFA, Sequential Organ Failure Assessment.

The 28-day mortality was 5.4% (*n* = 23/428) in the low presepsin group (≤755 pg/mL) and 24.2% (*n* = 79/327) in the high presepsin group (>755 pg/mL) (*p* < 0.001). The 28-day mortality was significantly different between the low presepsin group and the high presepsin group, even in subgroups including non-sepsis patients with delta SOFA < 2 (3.6% vs. 13.3%, *p* < 0.001) and patients with low lactate levels < 2 mmol/L (2.7% vs. 14.4%, *p* < 0.001) (Figure 3).

**Figure 4:**
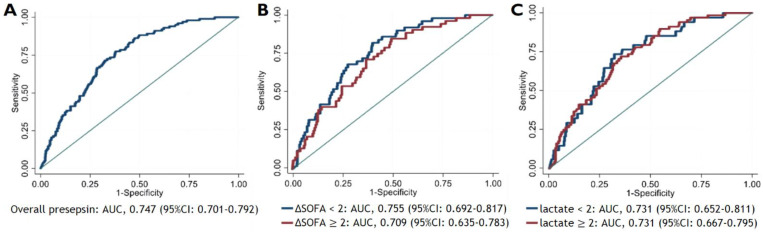
Area Under the Curve (AUC) of the Receiver Operating Characteristic (ROC) Curves of presepsin for predicting 28-day mortality according to the delta SOFA score and the lactate level in the following groups: (**A**) overall study population, (**B**) subgroups according to the delta SOFA (<2 or ≥2), and (**C**) subgroups according to the lactate levels (<2 mmol/L or ≥2 mmol/L).

The AUC of presepsin for predicting 28-day mortality was 0.747 (95% CI: 0.701–0.792). (Figure 4), which was significantly higher than that of PCT (AUC: 0.678, 95% CI: 0.630–0.725) (Supplementary Figure S1). The AUC was similar between non-sepsis patients with delta SOFA score < 2 (0.755, 95% CI: 0.701–0.792) and sepsis patients with delta SOFA ≥ 2 (0.709, 95% CI: 0.635–0.783). This trend was also consistent when the patients were divided according to the lactate level (<2 mmol/L vs. ≥2 mmol/L).

### 3.5. Combined Interpretation of the High Presepsin Cut-Off, Delta SOFA Score ≥ 2 Points, and Lactate ≥ 2 mmol/L

**Figure 5:**
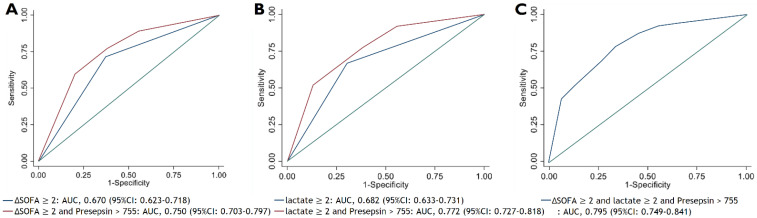
Area under the curve (AUC) of the receiver operating characteristic (ROC) curves of (**A**) delta SOFA score ≥ 2 points and combination model with high presepsin, (**B**) lactate ≥ 2 mmol/L and combination model with high presepsin, and (**C**) combination model of delta SOFA score ≥ 2, lactate ≥ 2 mmol/L, and presepsin. High presepsin was defined as a presepsin level greater than 755 pg/mL.

The AUC of presepsin with a cut-off value of 755 pg/mL in predicting 28-day mortality was 0.697 (95% CI: 0.653–0.742), which was the higher than that of delta SOFA score ≥ 2 (0.670 [95% CI: 0.623–0.718]) and lactate ≥ 2 mmol/L (0.682 [95% CI: 0.633–0.731]). When presepsin was combined with delta SOFA score and lactate individually, the AUC in predicting 28-day mortality was 0.750 (95% CI: 0.703–0.797) and 0.772 (95% CI: 0.727–0.818), respectively, showing a greater AUC than with exclusive use of each criterion (Figure 5). Combining the three criteria exhibited the greatest AUC of 0.795 (95% CI: 0.749–0.841).

The sensitivity and specificity of presepsin with a cut-off value of 755 pg/mL were 77.5% (95% CI: 68.1–85.1) and 62% (95% CI: 58.2–65.8), respectively (Table 3). If criteria for either presepsin or delta SOFA was fulfilled, greater sensitivity (89.2%, 95% CI: 81.5–94.5) was obtained compared to using the delta SOFA score alone. Positive criteria for both values increased specificity (79.9%, 95% CI: 76.7–82.9). Likewise, adding the lactate criterion or combining all three criteria showed similar trends. The highest sensitivity (94.1%, 95% CI: 87.6–97.8) was achieved when the three criteria were combined in an “or” manner; highest specificity (93.7%, 95% CI: 91.6–95.5) and PPV (51.8%, 95% CI: 40.7–62.7) were achieved with an “and” combination.

## 4. Discussion

Since presepsin was first discovered as a novel biomarker for sepsis in 2002 [13], a number of studies have demonstrated the significance of presepsin for identifying sepsis or predicting mortality. Although the diagnostic and prognostic values of presepsin for sepsis have been demonstrated in various studies [14,15,16,17], the complementary utility and applicability of presepsin in clinical practice still need further research.

Sepsis, a life-threatening organ dysfunction caused by infection, continues to be a major cause of death globally [18]. Multiple studies have established the advantage of prompt intervention, such as rapid antibiotics administration, in reducing mortality [19,20]. Early recognition of high-risk patients is crucial to improving patient outcomes. Nonetheless, it is often challenging to sort out high-risk patients in real-life clinical practice.

In this study, our data showed a significant difference in presepsin levels among the non-sepsis, sepsis, and septic shock patients, when categorized by the Sepsis-3 criteria. Regarding previous studies, the results of this study are in line with previous results on the prognostic value of presepsin. While the study was performed in a tertiary hospital with a cancer center, the study population comprised patients with malignancies, those who were immunocompromised, and those with multiple comorbidities. In addition, presepsin levels were significantly different between survivors and non-survivors in both the sepsis and non-sepsis groups. Thus, utilization of presepsin could be helpful in predicting the outcome in both sepsis and non-sepsis patients. Moreover, combining presepsin with other well-known markers, such as the Sepsis-3 definition by SOFA score and lactate, could provide greater predictive power. When presepsin was combined with delta SOFA score, the predictive power for mortality was greater than using SOFA score alone, suggesting the advantage of ancillary use of presepsin along with other markers. Thus, use of presepsin could improve the identification of high-risk patients.

Although the biological function of presepsin itself has yet to be elucidated, the primary source of presepsin secretion is CD14-positive monocyte/macrophage lineages which are triggered by bacterial phagocytosis [21]. Presepsin is currently known to rise faster than PCT and CRP [6,7,10]. It is also speculated that presepsin rises faster than lactate, considering the fact that increases in lactate level result from tissue hypoperfusion. Besides, presepsin shows excellent correlation between sample types including whole blood, and the result can be obtained within 17 min [11]. Hence, presepsin is a feasible marker for predicting mortality in sepsis, especially at an early stage before progression of organ failure. Utilization of presepsin may assist in identifying patients with potentially unfavorable outcomes and could improve mortality rates by facilitating prompt treatment.

Although other markers such as CRP and PCT have been widely used, those markers have their own limitations in terms of predicting prognosis [22]. Lactate levels could be also increased by hepatic dysfunction, renal dysfunction, or catecholamine, as well as sepsis-induced tissue hypoxia [23]. Compared with those biomarkers, presepsin might have some advantages in that it could be more specific for sepsis and could have an earlier increase. On the other hand, presepsin may be elevated in renal or hepatic dysfunction without infection [3,24] so further research would be needed for clinical interpretation.

There are several limitations to our study that deserve to be acknowledged. First, while the subjects were enrolled in a prospective manner, the selection criteria included procalcitonin and blood culture, which could cause selection bias. However, since procalcitonin and blood culture are generally ordered for almost all suspected sepsis patients, the chance of selection bias is relatively low. Besides, to the best of our knowledge, the number of reviewed subjects in this study is the largest among studies evaluating presepsin under the Sepsis-3 criteria, to date. Second, this study was conducted in a single institution, and approximately two-thirds of the enrolled subjects had a malignancy. Since the population of our study tended to be biased toward high severity, further research should be carried out to ascertain the prognostic value of presepsin in patients with less severe illness. Third, while various outcome measures for sepsis research have been proposed [25], only a single outcome measure, 28-day mortality, has been evaluated. Although 28-day mortality has been widely used as the primary measure representing the clinical outcome of sepsis, other patient-centered outcomes including long-term survival could potentially differ from 28-day mortality and further studies should investigate various sepsis outcomes to evaluate the clinical utility of presepsin [25]. Fourth, we were unable to obtain follow-up presepsin level changes over time. Since we only retrieved the presepsin level from a single time point at presentation, the actual onset of sepsis and whether the measured presepsin level represents the peak level are uncertain. Nonetheless, the characteristics of our data may better reflect the real-world clinical setting. Further research regarding the underlying mechanism of presepsin secretion, the biological role of presepsin, and the serial kinetics of presepsin according to the patient status are warranted.

## 5. Conclusions

Presepsin measurement had prognostic value for predicting 28-day mortality both in non-sepsis patients and sepsis patients. Complementary use of presepsin with the SOFA score of the Sepsis-3 clinical criteria or with the lactate level may identify more high-risk septic patients and provide useful prognostic information.

## Figures and Tables

**Table 1:** Baseline characteristics.

	Overall (*n* = 755)	Survivors (*n* = 653, 86.5%)	Non-Survivors (*n* = 102, 13.5%)	*p*-Value
**Age, years**	63.2 (51.9–72.1)	62.3 (49.8–72.1)	65.8 (59.8–71.7)	0.004
**Sex, male**	425 (56.3)	352 (54.0)	73 (71.6)	0.001
**Comorbidities**				
Diabetes	153 (20.3)	131 (20.1)	22 (21.6)	0.725
Cerebrovascular disease	63 (8.4)	57 (8.7)	6 (5.9)	0.334
Chronic cardiac disease	101 (13.4)	83 (13.5)	13 (12.8)	0.840
Chronic lung disease	41 (5.4)	34 (5.2)	7 (6.9)	0.492
Chronic liver disease	101 (13.4)	83 (12.7)	18 (17.7)	0.173
Chronic renal disease	74 (9.8)	66 (10.1)	8 (7.8)	0.472
Malignancy	497 (65.8)	409 (62.7)	88 (86.3)	<0.001
**Infection focus**				
Respiratory	185 (24.5)	138 (21.1)	47 (46.1)	<0.001
Urinary tract	117 (15.5)	107 (16.4)	10 (9.8)	0.087
Gastrointestinal	107 (14.2)	94 (14.4)	13 (12.8)	0.657
Hepatobiliary and pancreatic	101 (13.4)	88 (13.5)	13 (12.8)	0.840
Bone and soft tissues	66 (8.7)	62 (9.5)	4 (3.9)	0.063
Others	82 (10.9)	74 (11.3)	8 (7.8)	0.292
**Positive blood culture**	110 (14.6)	85 (13.0)	25 (24.5)	0.002
Gram-positive bacteria	35 (4.6)	27 (4.1)	8 (7.8)	0.098
Gram-negative bacteria	72 (9.5)	58 (8.9)	14 (13.7)	0.121
Fungus	5 (0.7)	1 (0.2)	4 (3.9)	<0.001
**SOFA score**	2 (1.0–5.0)	2 (1.0–4.0)	5 (3.0–9.0)	<0.001
**Patients with sepsis**	318 (42.1)	245 (37.5)	73 (71.6)	<0.001
**Patients with septic shock**	44 (5.8)	26 (4.0)	18 (17.7)	<0.001
**Laboratory tests**				
WBC (10^3^/μL)	7.8 (3.9–12.4)	7.7 (4.0–12.0)	9.3 (3.1–17.4)	0.030
Hemoglobin (g/dL)	10.4 (9.0–12.3)	10.5 (9.1–12.5)	9.5 (8.5–10.8)	<0.001
Platelet (10^3^/μL)	152 (75.0–242.0)	159 (84.0–245.0)	101 (36.0–226.0)	0.001
Total bilirubin (mg/dL)	0.8 (0.5–1.5)	0.7 (0.4–1.3)	1.3 (0.6–3.8)	<0.001
Creatinine (mg/dL)	0.8 (0.6–1.2)	0.8 (0.6–1.1)	1.1 (0.6–1.9)	0.001
Lactate (mmol/L)	1.5 (1.1–2.3)	1.4 (1.1–2.1)	2.4 (1.7–4.4)	<0.001
CRP (mg/dL)	6.8 (2.9–14.4)	6.4 (2.6–13.2)	11.2 (6.1–18.9)	<0.001
Procalcitonin (ng/mL)	0.4 (0.1–1.6)	0.3 (0.1–1.5)	1.0 (0.4–4.3)	<0.001
Monocytopenia	348 (46.1)	298 (45.6)	50 (49.0)	0.524

The data are presented as median (IQRs) for continuous variables or as numbers (%) for categorical variables. IQR, interquartile range; SOFA, Sequential Organ Failure Assessment; WBC, white blood cell count; CRP, C-reactive protein.

**Table 2:** Univariate and multivariable logistic regression analysis for 28-day mortality.

	Univariable			Multivariable		
	OR	95% CI	*p*-Value	OR	95% CI	*p*-Value
Presepsin > 755 pg/mL	5.61	3.44–9.16	<0.001	5.17	2.92–9.16	<0.001
Age, years	1.03	1.01–1.04	<0.001	1.02	1.01–1.04	0.016
Sex, male	2.15	1.36–3.39	<0.001	1.14	0.67–1.95	0.626
Malignancy *	3.75	2.09–6.74	<0.001	5.34	2.71–10.53	<0.001
Respiratory infection	3.19	2.07–4.91	<0.001	3.34	1.98–5.65	<0.001
Delta SOFA score ≥ 2	4.19	2.65–6.63	<0.001	2.83	1.65–4.83	<0.001
Lactate ≥ 2 mmol/L	4.60	2.95–7.17	<0.001	3.37	2.04–5.57	<0.001
Procalcitonin > 0.5 ng/mL	2.49	1.61–3.84	<0.001	0.99	0.57–1.71	0.967

* Malignancy refers to metastatic solid cancer or hematologic malignancy. *p*-value of goodness-of-fit with the Hosmer–Lemeshow test = 0.7595. OR odds ratio, CI confidence interval, SOFA Sequential Organ Failure Assessment.

**Table 3:** Prognostic accuracy of presepsin > 755 pg/mL, delta SOFA score ≥ 2 points, and lactate ≥ 2 mmol/L for predicting 28-day mortality.

	Sensitivity (95% CI)	Specificity (95% CI)	PPV (95% CI)	NPV (95% CI)
Presepsin > 755 pg/mL	77.5 (68.1–85.1)	62 (58.2–65.8)	24.2 (19.6–29.2)	94.6 (92–96.6)
ΔSOFA ≥ 2	71.6 (61.8–80.1)	62.5 (58.6–66.2)	23 (18.4–28)	93.4 (90.6–95.5)
Lactate ≥ 2 mmol/L	66.7 (56.6–75.7)	69.7 (66–73.2)	25.6 (20.4–31.2)	93.0 (90.4–95.1)
Presepsin > 755 pg/mL or ΔSOFA ≥ 2	89.2 (81.5–94.5)	44.6 (40.7–48.5)	20.1 (16.5–24.1)	96.4 (93.6–98.2)
Presepsin > 755 pg/mL or lactate ≥ 2 mmol/L	92.2 (85.1–96.6)	44.6 (40.7–48.5)	20.6 (17–24.6)	97.3 (94.8–98.8)
Presepsin > 755 pg/mL or ΔSOFA ≥ 2 or lactate ≥ 2 mmol/L	94.1 (87.6–97.8)	34.3 (30.7–38.1)	18.3 (15.1–21.9)	97.4 (94.4–99)
Presepsin > 755 pg/mL and ΔSOFA ≥ 2	59.8 (49.6–69.4)	79.9 (76.7–82.9)	31.8 (25.3–38.9)	92.7 (90.3–94.7)
Presepsin > 755 pg/mL and lactate ≥ 2 mmol/L	52.0 (41.8–62.0)	87.1 (84.3–89.6)	38.7 (30.5–47.4)	92.1 (89.7–94.1)
Presepsin > 755 pg/mL and ΔSOFA ≥ 2 and lactate ≥ 2 mmol/L	43.1 (33.4–53.2)	93.7 (91.6–95.5)	51.8 (40.7–62.7)	91.3 (89.0–93.4)

Δ, delta; SOFA, Sequential Organ Failure Assessment; CI, confidence interval; PPV, positive predictive value; NPV, negative predictive value.

## Data Availability

The datasets used and/or analyzed during the current study are available from the corresponding author upon reasonable request.

## Supplementary Materials

The following are available online at https://www.mdpi.com/article/10.3390/biomedicines9091076/s1, Supplementary Table S1: Clinical characteristics of the patients with non-sepsis, sepsis, and septic shock, Supplementary Figure S1: Comparisons of the receiver operating characteristic (ROC) curves of presepsin and procalcitonin for predicting 28-day mortality: (**a**) overall group, (**b**) non-sepsis patients, and (**c**) sepsis patients.

## Abbreviations

ROC	Receiver operating characteristic
AUC	Area under the curve
IQR	Interquartile range
SOFA	Sequential organ failure assessment
PCT	Procalcitonin
CRP	C-reactive protein
LPS	Lipopolysaccharide
ED	Emergency department
ICU	Intensive care unit
PST	Plasma separating tube
ALP	Alkaline phosphatase
QC	Quality control
CV	Coefficient of variation
CI	Confidence interval
MAP	Mean arterial pressure
PPV	Positive predictive value
NPV	Negative predictive value
COVID-19	Coronavirus disease 2019
OR	Odds ratio
aOR	Adjusted odds ratio
