# Supplementary material for: Complementary Use of Presepsin with the Sepsis-3 Criteria Improved Identification of High-Risk Patients with Suspected Sepsis
Source: Biomedicines. 2021 Aug 24;9(9):1076. doi: 10.3390/biomedicines9091076 (PMC8469631; doi:10.3390/biomedicines9091076)

Supplementary Table S1. Clinical characteristics of the patients with non-sepsis, sepsis, and septic shock

	**Non-Sepsis (*n* = 437, 57.9%)**	**Sepsis (*n* = 274, 36.3%)**	**Septic shock (*n* = 44, 5.8%)**	***p*-value**
**Age, years**	61.2 (47.7-70.2)	65.1 (54.5-73.6)	68.0 (57.1-73.9)	<0.001
**Sex, male**	221 (50.6)	179 (65.6)	25 (56.8)	<0.001
**Comorbidities**				
Diabetes	89 (20.4)	50 (20.1)	9 (20.5)	0.995
Cerebrovascular disease	32 (7.3)	27 (9.9)	4 (9.1)	0.486
Chronic cardiac disease	55 (12.6)	40 (14.6)	6 (13.6)	0.744
Chronic lung disease	18 (4.1)	18 (6.6)	5 (11.4)	0.075
Chronic liver disease	53 (12.1)	40 (14.6)	8 (18.2)	0.403
Chronic renal disease	38 (9.8)	29 (10.6)	2 (4.6)	0.457
Malignancy	296 (67.7)	171 (62.4)	30 (68.2)	0.327
**Infection focus**				
Respiratory	90 (20.6)	82 (29.9)	13 (29.6)	0.014
Urinary tract	74 (16.9)	37 (13.5)	6 (13.6)	0.441
Gastrointestinal	60 (13.7)	39 (14.2)	8 (18.2)	0.722
Hepatobiliary and pancreatic	44 (10.1)	51 (18.6)	6 (13.6)	0.005
Bone and soft tissues	46 (10.5)	18 (6.6)	2 (4.6)	0.114
Others	49 (11.2)	24 (8.8)	9 (20.5)	0.064
**SOFA score at enrollment***	1 (0-2)	4 (3-6)	9 (6-11.5)	<0.001
**Laboratory tests**				
Lactate (mmol/L)	1.4 (1.1-2.0)	1.6 (1.1-2.3)	4.4 (2.5-8.1)	0.060
Lactate ≥2 (mmol/L)	124 (28.4)	98 (35.8)	44 (100)	<0.001
CRP (mg/dL)	5.9 (2.4-11.1)	9.2 (4.2-17.1)	8.3 (4.9-24.0)	<0.001
Procalcitonin (ng/mL)	0.2 (0.1-0.7)	0.8 (0.2-2.5)	7.0 (1.7-45.3)	<0.001
Procalcitonin > 0.5 (ng/mL)	140 (32)	166 (60.6)	37 (84.1)	<0.001
Presepsin (pg/mL)	466 (284-938)	933 (472-1861)	1179 (642.5-3224.5)	<0.001
With monocytopenia	441.5 (266-937)	1054 (505-1906)	1246.5 (732-3181)	0.028
Without monocytopenia	503 (293-973)	882 (451-1583)	880 (511-3512)	<0.001
Presepsin > 755 (pg/mL)	135 (30.9)	162 (59.1)	30 (68.2)	<0.001
**Positive blood cultures**	37 (8.5)	49 (17.9)	23 (52.3)	<0.001
**28-day mortality**	29 (6.6)	55 (20.1)	18 (40.9)	<0.001

The data are presented as median (IQRs) for continuous variables or as numbers (%) for categorical variables. $p < 0.05$ was corrected by Bonferroni's method.

IQR, interquartile range; SOFA, Sequential Organ Failure Assessment; CRP, C-reactive protein.

*SOFA score at enrollment: within 24 hours after ED presentation.

Supplementary Figure S1. Comparisons of the receiver operating characteristic (ROC) curves of presepsin and procalcitonin for predicting 28-day mortality: (A) overall group, (B) non-sepsis patients, and (C) sepsis patients

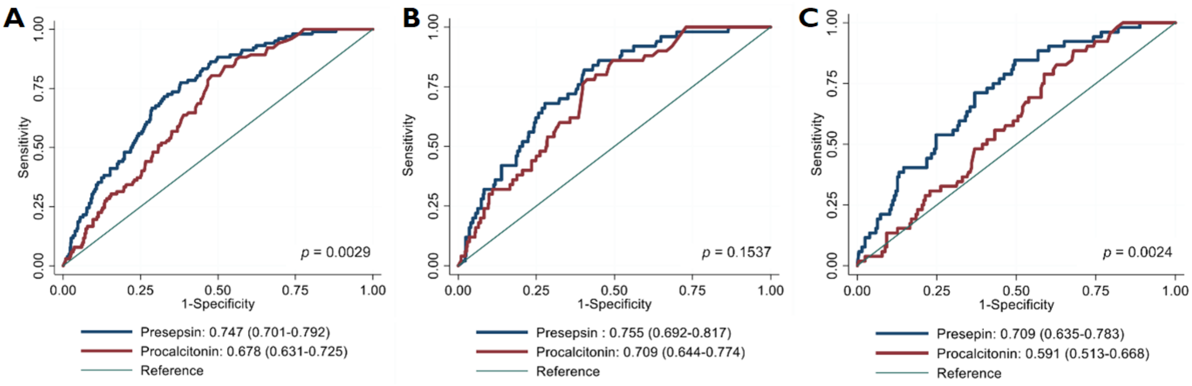

Supplement: Supplementary file 1 [file biomedicines-09-01076-s001.zip › biomedicines-1313514-supplementary.pdf]
